# Post-attempt care in borderline personality disorder: effects of safety planning, crisis response, caring contacts and assertive follow-up

**DOI:** 10.1192/j.eurpsy.2026.10685

**Published:** 2026-07-31

**Authors:** D. Jelaga, M. Belous

**Affiliations:** Department of Mental Health, Medical Psychology and Psychotherapy, Nicolae Testemițanu State University of Medicine and Pharmacy, Chisinau, Moldova, Republic of

## Abstract

**Introduction:**

BPD has a high risk of repeat suicidal behaviour peaking in the first 30 days post attempt. Scalable components such as safety planning structured crisis response caring contacts and assertive follow up are used yet effect sizes in BPD remain uneven.

**Objectives:**

To quantify associations with re attempts adherence and service use; to compare adolescents versus adults and acute emergency department or inpatient versus community settings; and to identify predictors and implementation signals.

**Methods:**

Systematic searches in MEDLINE/PubMed, Embase, PsycINFO and Web of Science (2020-2025). Designs: randomised trials, cohort/longitudinal, observational/ecological, and meta-/systematic reviews. Exclusions: no distinct BPD data/off-topic/non-suicidality outcomes. Flow: 642 records; after deduplication and screening, 92 full texts; 34 included; 58 excluded (no BPD=26; off-topic=18; insufficient=14). Populations: adolescents 13–18 years; adults (means 30–40 years); ~74% female adults. Settings: emergency department, brief admission, community. Outcomes: re-attempts (3/6/12 months), adherence (≥1 follow-up/therapy), ED re-presentations/hospitalisations; pp=percentage points; adverse events were rarely reported.

**Results:**

Most estimates were transdiagnostic; BPD subgroups were small yet directionally consistent. Safety planning (observational, ED vs usual care): adults 5% to 3% re-attempts at 6 months (−2 pp; −40–45% relative) and ~+100% linkage to ambulatory care; adolescents ~0% aggregate effect when delivered alone. Crisis response: an individualised crisis-response plan (randomised vs safety-as-usual/contract) was associated with 19% to 5% at 6 months (−74%) and fewer hospital days. Brief admission for BPD: mixed randomised findings; observationally, self-harm 17% to 8% (−53%) and ED presentations 56% to 8% (−86%) at 3 months. Caring contacts: meta-analyses suggest −20–40% re-attempts at 6–12 months; one randomised texting study 14.9% to 9.0% (−40%) at 12 months; large-scale roll-out increased service use ~10–19%, effects on attempts variable. Assertive follow-up/tele-monitoring: multifactorial aftercare associated with −31–38% 12-month recurrence; a 12-month tele-follow-up programme 41.4% to 21.4% (−54%) with longer time-to-event. Predictors: in outreach cohorts, BPD diagnosis ≈+80% odds of re-attempt (OR≈1.8).

**Conclusions:**

A standardised post-attempt pathway (safety plan at discharge + contact within 24–72 h + periodic caring contacts + assertive follow-up for 6–12 months) was associated with ~30–50% lower recurrence in adults and better adherence. For adolescents, safety planning alone appears insufficient; family-integrated, BPD-specific care is advisable. Major limitation: generalisability is constrained by heterogeneous contact “dose”/fidelity and the scarcity of BPD-only RCTs; protocol standardisation and longer follow-up are warranted.

**Disclosure of Interest:**

None Declared

